# Supplementary material for: Causal interplay between lactose intolerance and gut microbiota: a combined bidirectional Mendelian randomization and in vivo validation study
Source: Front Nutr. 2026 Jun 1;13:1803337. doi: 10.3389/fnut.2026.1803337 (PMC13265576; doi:10.3389/fnut.2026.1803337)
Supplement: Supplementary file 1 [file Data_Sheet_1.zip › supplementary materials/Forward/leave one out cross validation/ebi-a-GCST90027467.finngen_R12_E4_LACTONAS.pdf]

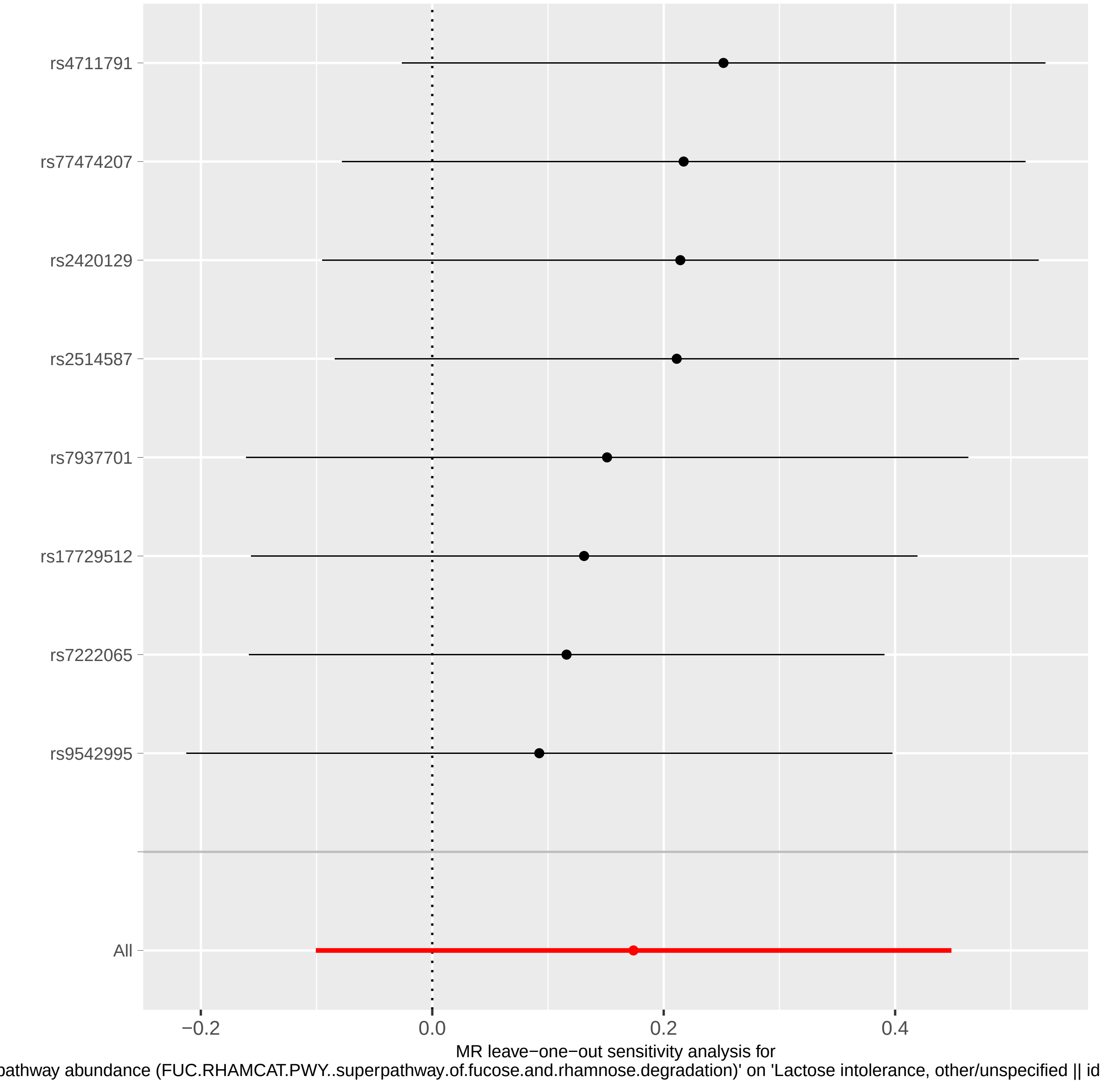
rs4711791
rs77474207
rs2420129
rs2514587
rs7937701
rs17729512
rs7222065
rs9542995
All
−0.2
0.0
0.2
0.4
MR leave−one−out sensitivity analysis for
pathway abundance (FUC.RHAMCAT.PWY..superpathway.of.fucose.and.rhamnose.degradation)' on 'Lactose intolerance, other/unspecified || id
